# On the Road to Strain-Resolved Comparative Metagenomics

**DOI:** 10.1128/mSystems.00190-17

**Published:** 2018-03-13

**Authors:** Nicola Segata

**Affiliations:** a Centre for Integrative Biology, University of Trento, Trento, Italy

**Keywords:** metagenomics, human microbiome, computational biology, comparative microbial genomics, strain profiling

## Abstract

Metagenomics has transformed microbiology, but its potential has not been fully expressed yet. From computational methods for digging deeper into metagenomes to study designs for addressing specific hypotheses, the Segata Lab is pursuing an integrative metagenomic approach to describe and model human-associated microbial communities as collections of strains. Linking strain variants to host phenotypes and performing cultivation-free population genomics require large cohorts and meta-analysis strategies to synthesize available cohorts but can revolutionize our understanding of the personalized host-microbiome interface which is at the base of human health.

## Abstract (precis)

Metagenomics has transformed microbiology, but its potential has not been fully expressed yet. From computational methods for digging deeper into metagenomes to study designs for addressing specific hypotheses, the Segata Lab is pursuing an integrative metagenomic approach to describe and model human-associated microbial communities as collections of strains.

## PERSPECTIVE

Metagenomics (1) is typically considered a holistic top-down approach for studying microbiomes; by profiling the overall taxonomic composition and functional potential of a metagenome, it is possible to describe the microbiome as an ecological entity. However, the building blocks of a microbial community are its single microbial strains. Because different strains in the same microbial species can be substantially different, I argue that a reductionist bottom-up approach is necessary to model and understand the complexity of microbial communities.

The reductionist approach of profiling the strain as the fundamental unit of the microbiome from metagenomics is, however, hampered by limitations in computational analysis. My laboratory focuses on solving these challenges to allow characterizing strains in human metagenomes with the same level of resolution as is available for single-isolate sequencing. This would bring the field into the next generation of microbiome studies.

I discuss the key questions that, in my opinion, need to be answered in the next few years to exploit the potential of shotgun metagenomics in fields of medicine, ecology, and microbiology.

## DO WE NEED STRAIN-LEVEL RESOLUTION FOR MICROBIOME RESEARCH?

Pregenomic and isolate sequencing studies of (opportunistic) pathogens provide overwhelming evidence that many microbial phenotypes are strain specific. The species *Escherichia coli* includes strains that are gut commensals as well as highly pathogenic (2) and carcinogenic (3). Different strains of *Helicobacter pylori* are associated with widely different risks for gastric cancer (4). These are just two examples provided by infectious microbial genomics. Similar evidence for normal members of the human microbiome is growing but remains anecdotal; some but not all intestinal strains of *Eggerthella lenta* can inactivate a cardiac drug (5), some subtypes of *Prevotella copri* are associated with increased risk for rheumatoid arthritis (6) whereas others are associated with healthy and low-fat diets, and some variants of *Staphylococcus epidermidis* seem to be associated with psoriatic skin (7). Because massive strain-level heterogeneity in the human microbiome has already been observed (8, 9), I argue that systematically characterizing these variations with respect to conditions of interest, including diseases, would provide an unprecedented tool to generate hypotheses on mechanistic host-microbiome interactions and microbial targets for diagnostic and therapeutic strategies.

## WHAT ARE MICROBIAL STRAINS?

There are currently no strict definitions of what a bacterial or archaeal strain is, and this inevitably causes difficulties and misunderstandings. If we were to strictly link a strain with its genome, single-nucleotide variants (SNVs) accumulating over microbial generations in pure cultures would lead to calling a given organism a different strain over time. Moreover, the number of different strains would be too large to be tractable, and errors in high-throughput sequencing would make strain identity not testable. On the other hand, any threshold applied to the number of nucleotide variants allowed between “same” strains would be an arbitrary cutoff, would be suboptimal in most cases, and would not account for events such as gene loss/gain or horizontal gene transfer.

It would be ideal to define strains as microbial entities that, despite a limited genetic heterogeneity, have the same phenotype under different conditions. However, this would be impossible to establish in practice. Phylogenetic modeling of single microbial genomes can overcome the need for a strict definition in some cases. But because synonymous mutations and other genetic variations that do not give any evolutionary or ecological (dis)advantages can be present in the same microbiome sample, it is not even clear whether we should talk about strain populations or strain clouds instead of single strains. Proposing a general definition of a strain is outside the scope of this Perspective, but it will be crucial for this line of research to at least define practical and operational definitions for strains.

## CAN WE PROFILE MICROBIOMES WITH STRAIN-LEVEL RESOLUTION?

We have recently developed three complementary methods to extract strain-level signatures from metagenomes. They are all based on popular methods used to characterize strains from pure cultures adapted to the complexity of metagenomes and the presence of confounding reads. MetaMLST (10) exploits a few species-specific hypervariable loci previously defined mostly for (opportunistic) pathogens and can rely on several thousands of already characterized strain profiles. With StrainPhlAn (9), we brought to metagenomics the single-nucleotide variant (SNV) analysis that is routinely applied to the core genome of sequenced microbial isolates. This tool uses millions of reference sequences identified offline (11) as unambiguous clade-specific marker genes (a subset of the core genes) and infers SNVs and phylogenetic relations across strains in different samples. PanPhlAn (9), in contrast, defines strains as unique combinations of genes in the pangenome of a species and can thus associate genes, operons, and functions with specific sets of strains.

Some results produced by these methods include discovery of the strain-level tropism of *Neisseria* in different locations of the oral cavity (12), the intestinal *E. coli* subtypes associated with necrotizing enterocolitis in preterm newborns (13), and the strain heterogeneity in the skin microbiome in psoriasis (7). These examples confirm that strain-resolved metagenomics not only is possible but also can generate otherwise-unattainable insights from microbiome studies.

Other groups are also developing tools for strain profiling from metagenomics, and it is important for the field that complementary and independent approaches are implemented. The DESMAN pipeline (14) exploits coassembly and binning to identify haplotypes and resolve them into strains, whereas MetaSVN (15) directly calls SNVs on metagenomes mapped against reference genomes to estimate allele frequencies, and ConStrains (16) adopts the MetaPhlAn2 (11) database of StrainPhlAn to bin marker gene variations into strain groups. Several challenges still need to be addressed, including profiling nondominant strains of low abundance and avoiding chimeric strain reconstruction. Our current efforts are focused on improved approaches to exploit the rapidly expanding information available from reference genomes and the effective combination of metagenomic assembly with SNV-based and pangenome-based profiling.

## CAN WE TRACK AND COMPARE STRAINS ACROSS SAMPLES AND SUBJECTS?

Genetic signatures of strains extracted from metagenomes need to be compared across samples to enable comparative genomics. However, strains from metagenomes are likely to have lower quality than genomes from isolate sequencing, and it can thus be difficult to disentangle true strain variability from reconstruction inaccuracies. Although validation using synthetic and semisynthetic data suggested that our methods perform well for comparative genomics, real data were crucial to confirming it. For example, all three methods accurately profiled the pathogenic *E. coli* strain in metagenomes from the German outbreak in 2011 (17) for which isolate sequencing and targeted metagenomic assembly of the pathogen are available.

Strain comparison across metagenomes also highlighted that, for the majority of species, only a few SNVs and variable genes are detected in strains recovered from the gut metagenomes of an individual sampled over few months (9, 18). Intersubject strain variation is instead orders of magnitude larger both in the gut and in the oral microbiome (12), further confirming the precision of the methods and suggesting that each of us has a unique microbiome at the strain level.

This uniqueness of microbial strains in each individual is functional for tracking strains across subjects. This was exploited by Li and colleagues for detailing strain engraftment following fecal transplantation (19) and by our group in describing transmission of microbiome members from mothers to their infants during the first weeks of life (20). It is thus already possible to compare strain profiles across samples, but additional work is needed to model strain variation with respect to the level of underlying between-subject variability and intrasubject evolution.

## CAN WE SCALE STRAIN PROFILING TO THOUSANDS OF MICROBIOMES?

Comparative genomics becomes more effective as the numbers of genomes being compared increase. As of September 2017, the number of human-associated metagenomes in public repositories exceeded 20,000, and the rate at which new metagenomes are being deposited is still increasing. It is thus imperative for strain profiling to be applicable to thousands of metagenomes. Despite the computational infrastructure needed to handle thousands of metagenomes, our methods can be applied, in principle, to the whole number of available metagenomes. Metagenomic assembly is, in contrast, more difficult to scale to such a number of samples, especially in performing coassembly of all of the elements in the whole data set, but we believe that there is still room for substantial improvement in both reference-free and assembly-based approaches.

## CAN WE USE STRAIN PROFILING FOR CULTIVATION-FREE MICROBIAL POPULATION GENOMICS?

Genetic signatures of strains are not directly affected by biases such as those represented by differences in sample collection and DNA extraction that impact the quantitative estimation of species or gene abundances in metagenomes. Combined with the scalability of strain profiling, this provides a tantalizing opportunity to perform strain-level comparative genomics and epidemiology of underinvestigated and hard-to-cultivate strains reconstructed from the whole set of available human metagenomes. Indeed, we showed strong associations between strain variation and biogeographical patterns (9, 21), with species such as the highly prevalent *Eubacterium rectale* divided into subspecies structures specific to different continents. And the possibility of performing population genomics and biogeography analysis for hundreds of microbial species is not limited to bacteria. By analyzing 2,154 metagenomes, we have also investigated *Blastocystis* (22), an overlooked intestinal eukaryotic parasite, which we found in a relatively large fraction of the population (~15%) and with increased prevalence in healthy individuals compared to subjects with disease conditions ranging from Crohn’s diseases to colorectal cancer.

Comprehensive meta-analyses of metagenomic data sets for population genomics require, however, standardization of raw sequencing data and of the information associated with the samples (metadata). Because Illumina sequencing is the de facto standard for metagenomics and because presequencing biases affect strain profiling only minimally, our experience is that it is feasible for a laboratory with sufficient computational infrastructure to process public metagenomes by automatically downloading the raw sequences and running them through strain profilers. For a number of practical and technical reasons, the retrieval of consistent metadata is much more challenging. Manual checking and curation of metadata appear to be the only solution to enable cross-study sample analysis, and we organized our manual curation effort into the curatedMetagenomicData resource (23). This is a constantly updated data package readily providing, through R (or the command line), precomputed microbiome profiles for many thousands of human microbiome samples from dozens of studies and host conditions. Because of the difficulties encountered in manual curation of metagenomic metadata, we have implemented a syntax-checking system and are trying to engage the community and the data set producers in this curation effort.

## CAN WE IDENTIFY STRAIN-LEVEL VARIANTS ASSOCIATED WITH HOST CONDITIONS?

Microbial traits such as virulence or antibiotic resistance in (opportunistic) pathogens have all been linked with strain-specific genomic characteristics using cultivation-based studies. We hypothesize that the same principles also apply to interaction between members of the human microbiome and our bodies. We found preliminary evidence of strain-level adaptation of *Staphylococcus epidermidis* to psoriasis-affected skin (7), and we are generating similar hypotheses for intestinal and oral bacteria associated with colorectal cancer and periodontitis.

Because the overall intersubject strain-level variability of a microbiome is extremely large, similarly large sample sizes are needed to obtain strong statistical support for strain-level associative multiple hypothesis testing. Resources such as the already mentioned curatedMetagenomicData resource (23) are crucial not only to enlarge the sample size but also to stratify hypotheses by conditions such as age, geography, and diet. By combining newly produced metagenomic data with publicly available samples, we are aiming to find specific genes, sequence variants, operons, and genomic islands associated with enhanced risk for human diseases.

## CAN WE RECONSTRUCT STRAINS FROM UNCHARACTERIZED ORGANISMS?

A considerable fraction of the human microbiome remains hidden from reference-based profiling. Although genomes for new species are being published more frequently, metagenomic assembly offers the potential of reference-free microbial identification. While algorithms able to generate high-quality genomic contigs exist (24, 25), it is more challenging to bin contigs into consistent genomes of the strains present in a microbiome (26), even with expert supervision (27, 28). Binning and taxonomic classification are also hampered by inconsistencies in the microbial taxonomy and by mislabeling in deposited sequence data. We believe that a comprehensive survey of the uncharacterized microbially diverse populations in metagenomes (including viral organisms and mobile elements) should be performed by relying only on very-high-quality metagenomically assembled bins and by contextualizing them through phylogenetic rather than taxonomic approaches. Our new developments of PhyloPhlAn (29) are going in this direction, but metagenomic characterization of unknown genomes in the human microbiome remains an open challenge that is necessary to obtain a comprehensive strain-level description of microbial communities.

## CONCLUSIONS

I have outlined the key questions that I think we need to address on the road to empowering metagenomics with the resolution needed to generate reductionist and mechanistic hypotheses on the link between the human microbiome and human health. In my laboratory and with the multidisciplinary network of collaborations that we maintain, we pursue these goals by developing tools to accurately profile strains from metagenomes and by scaling strain profiling to many thousands of metagenomes with manually curated metadata.
